# Alterations in Gut Microbiota of Infants Born to Mothers with Obesity

**DOI:** 10.3390/biomedicines13040838

**Published:** 2025-03-31

**Authors:** Zarina Meiirmanova, Nurislam Mukhanbetzhanov, Zharkyn Jarmukhanov, Elizaveta Vinogradova, Saniya Kozhakhmetova, Marina Morenko, Arailym Duisebayeva, Dimitri Poddighe, Almagul Kushugulova, Samat Kozhakhmetov

**Affiliations:** 1 Laboratory of Microbiome, Center for Life Sciences, National Laboratory Astana, Nazarbayev University, 53 Kabanbay Batyr Ave., Block S1, Astana Z05H0P9, Kazakhstan; zarina.meiirmanova@gmail.com (Z.M.); nurislam.mukhanbetzhanov@nu.edu.kz (N.M.); zharkyn.jarmukhanov@nu.edu.kz (Z.J.); st.paulmississippi@gmail.com (E.V.); arailym.kudabaikyzy@gmail.com (A.D.); akushugulova@nu.edu.kz (A.K.); 2 Department of Children’s Diseases with Courses in Allergology, Hematology and Endocrinology, NJSC “Astana Medical University”, Astana Z01G6C5, Kazakhstan; morenko.m@amu.kz; 3 Interdisciplinary Sports Research, Center for Genetics and Life Sciences, Sirius University of Science and Technology, 1 Olympic Ave., Sirius Federal Territory 354340, Russia; 4 National Center for Biotechnology, Astana Z05K8D5, Kazakhstan; saniya.s.kozhakhmetova@gmail.com; 5 Innovative Center ArtScience, Astana Z11F5A9, Kazakhstan; 6 College of Health Sciences, VinUniversity, Gia Lam District, Hanoi 10000, Vietnam; dimitri.p@vinuni.edu.vn; 7 Kazakhstan Society of Human Microbiome Researchers, Astana Z05H0P9, Kazakhstan

**Keywords:** maternal obesity, infant gut microbiome, shotgun metagenomics, antibiotic resistance genes, early-life microbiota, infant health

## Abstract

**Background:** The impact of maternal obesity on offspring health remains a major and pressing issue. We investigated its impact on the development of the infant gut microbiome during the first six months of life, examining the taxonomic composition, metabolic pathways, and antibiotic resistance genes. **Methods:** Twenty-four mother–infant pairs were divided into maternally obese (OB, BMI > 36) and normal weight (BM) groups. Shotgun metagenomic sequencing was performed on stool samples collected at birth and at 1, 3, and 6 months. A total of 12 maternal samples and 23 infant samples (n = 35) in the obese group and 12 maternal samples and 30 infant samples (n = 42) in the control group were sequenced. The analysis included taxonomic profiling (MetaPhlAn 4), metabolic pathway analysis (HUMAnN 3), and antibiotic resistance gene screening (CARD/ABRicate). **Results:** The OB group showed reduced alpha diversity in the first month (*p* ≤ 0.01) and an increased *Firmicutes/Bacteroidetes* ratio, peaking at 3 months (*p* ≤ 0.001). The metabolic profiling revealed enhanced carbohydrate breakdown (*p* ≤ 0.001) in the BM group and lipid biosynthesis (*p* ≤ 0.0001) in the OB group pathways. Strong correlations emerged between *Lactobacillales* and fatty acid biosynthesis (r = 0.7, *p* ≤ 0.0001) and between *Firmicutes* and lincosamide (r = 0.8, *p* ≤ 0.0001). **Conclusions:** The infants of obese mothers had significantly altered development of the infant gut microbiome, affecting both composition and metabolic potential. These changes may have long-term health consequences and suggest potential therapeutic targets for intervention.

## 1. Introduction

The increasing prevalence of maternal obesity is emerging as a significant public health concern due to its potential impact on offspring health. Recent studies have demonstrated that maternal obesity and excess weight are associated with substantial alterations in infant intestinal microbiome composition, particularly among vaginally delivered children from families with higher socioeconomic status [1,2]. Basak et al. (2022), in their review, demonstrated that maternal obesity and gut microbiota are closely associated with neurodevelopment, whereby alterations in the a mother’s microbiota composition influence the development and function of the gastrointestinal tract of her offspring [3].

The human gut microbiome plays a crucial role in maintaining metabolic health. During pregnancy, maternal obesity alters the structure and composition of the gut microbiota, characterized by an increase in the relative abundance of *Firmicutes* and depletion of *Proteobacteria* [4]. These microbial changes may be transmitted to the infant, eventually influencing colonization and increasing the risk of metabolic disorders. Multiple factors may influence this process, including maternal pre-pregnancy body mass index, gestational weight gain, mode of delivery, and breastfeeding practices [5,6].

Furthermore, according to Guzzardi et al. (2022), maternal pre-pregnancy overweight and neonatal gut bacterial colonization are significantly associated with cognitive development in pre-school-age offspring. Their longitudinal study revealed that maternal overweight leads to the depletion of beneficial bacteria such as *Bifidobacterium*, *Blautia*, and *Ruminococcus*, and correlates with reduced practical reasoning scores in offspring at 36 months of age. These findings indicate the influence of maternal metabolic status on both the gut microbiota composition and cognitive function in early childhood [7].

Research over the past decade has significantly expanded our understanding of microbial colonization processes in the infant gut. There are suggestions that microbial colonization of the infant intestine begins in utero through amniotic fluid [8]. This shifts the traditional paradigm of a microbe-free intrauterine environment and emphasizes the importance of maternal microbiota at the prenatal stage. Furthermore, children born to mothers with obesity exhibit significant differences in gut microbiome composition, including changes in specific bacterial taxa such as *Faecalibacterium, Eubacterium*, and *Blautia*. These microbial modifications may influence the development of the infant’s immune system, metabolism, and appetite regulation [9]. As noted by Gohir et al. (2015), the gut microbiome of obese mothers is associated with inflammatory processes and increased risk of obesity in offspring [10].

In recent years, interest in the relationship between maternal obesity and the infant microbiome has increased significantly. Buffington et al. (2016) demonstrated that maternal obesity induced by a high-fat diet impacts the microbiota structure and may contribute to behavioral disorders associated with alterations in oxytocin transmission in the ventral tegmental area [11]. The authors emphasize the potential significance of the microbiome in the development of neurological and behavioral aspects related to maternal obesity. Meanwhile, Denizli et al. (2022) showed in their review that maternal obesity may program long-term changes in inflammatory and immune responses in offspring. These changes are potentially associated with an increased risk of developing chronic diseases, including metabolic syndrome [12].

Chu et al. (2016) discovered that the composition and structure of the neonatal gut microbiome varied significantly depending on maternal pre-pregnancy weight, with infants born to overweight mothers exhibiting reduced *Bacteroides* diversity and increased levels of pro-inflammatory taxa [13]. These differences in early-life microbial communities may have long-term implications for metabolic programming and disease susceptibility.

Recent investigations have unveiled potential mechanisms underlying the influence of maternal obesity on infant microbiome. Soderborg and colleagues (2018), conducting microbiota transplantation from the offspring of obese mothers, found that maternal obesity is associated with alterations in the gut microbiota metabolic pathways in offspring, potentially contributing to increased intestinal permeability and metabolic dysfunction [14], suggesting a causal role of the altered microbiome in programming metabolic health. Ferretti et al. (2018) identified, through studying the longitudinal dynamics of the infant microbiome during the first year of life, that BMI and other maternal factors have a prolonged influence on infant microbiome development, which is particularly noticeable in the first months of life [15].

In the context of metabolic programming, Hasan et al. (2018) identified specific bacterial taxa that were differentially represented in the offspring of mothers with gestational diabetes 5 years after birth. Specifically, elevated levels of the genus *Anaerotruncus*, associated with glucose intolerance, indicate a potential link between maternal metabolic disturbances and long-term changes in the offspring microbiome [16]. Furthermore, maternal obesity may significantly affect lipid biosynthesis pathways in infants, with studies indicating changes in gene expression related to mitochondrial and lipid metabolism in the umbilical vein endothelial cells of infants [17]. These changes, in conjunction with restructuring in the intestinal microbiome, suggest a complex interaction between maternal metabolic status and infant development, potentially contributing to the intergenerational transmission of obesity risk [18].

Given the growing body of information indicating the importance of early gut colonization for future child health and development, there is an urgent need for a deeper understanding of the impact of maternal obesity on infant microbiome formation. The aim of this research is to investigate age-specific alterations in infant gut microbiota composition associated with maternal obesity, with a particular focus on the dynamics of taxonomic composition, metabolic pathways, and antibiotic resistance genes during early postnatal development.

## 2. Materials and Methods

### 2.1. Subject Recruitment

This prospective study included 24 mothers and their infants, recruited between 2021 and 2023 at Perinatal Center No. 2 in Astana. The study included volunteers who provided informed consent for themselves and their infants. The exclusion criteria comprised prematurity, the presence of congenital pathology in infants, and antimicrobial use during the last trimester of pregnancy. Delivery mode (vaginal birth vs. Cesarean section) was documented for all mother–infant pairs. Sample collection was conducted in several stages: neonatal meconium was collected by specially trained personnel, with subsequent samples collected by research pediatricians during scheduled visits at 1, 3, and 6 months after birth. Each visit included anthropometric measurements (weight, height) and data collection on feeding methods, over-the-counter medication use, and the timing of formula and complementary food introduction. Infants who received antibiotic therapy were excluded from the study. Two observational groups were formed based on maternal body mass index: the main group (BMI > 36, with obesity, OB) and the control group (BM, BMI < 32).

### 2.2. DNA Extraction from Feces

Post-defecation, the meconium and fecal samples were immediately placed in tubes containing DNA/RNA stabilizing solution (DNA/RNA Shield Collection Tube, Zymo Research, R1101, Irvine, CA, USA) and stored and transported at +4 °C until DNA isolation. Total DNA was extracted using the ZymoBIOMICS DNA Miniprep Kit (Zymo Research, D4300, Irvine, CA, USA), with sterile µQ water as a negative extraction control. The extracted DNA quality was assessed spectrophotometrically (OD260/280 ratio) using Nanodrop and by electrophoresis in 1.0% agarose gel. The concentration and quality of the purified DNA samples were determined using a Qubit 3.0 fluorometer (Thermo Fisher Scientific, Waltham, MA, USA). Sequencing was performed at the Novogene laboratory (Beijing, China) on the Illumina NovaSeq 6000 platform following the manufacturer’s standard protocols.

### 2.3. Measuring Fecal pH

The fecal samples collected in the DNA/RNA Shield Collection Tubes (Zymo Research, R1101, Irvine, CA, USA) were thawed at room temperature before analysis. pH measurements were performed using an Orion Star™ A214 pH/ISE Benchtop Meter (Thermo Scientific™, Waltham, MA, USA).

### 2.4. Data Analysis and Statistics

Primary sequencing data analysis was conducted using the bioBakery 3 integrated methods suite for taxonomic, functional, and phylogenetic profiling of metagenomes. The taxonomic profiling was performed using MetaPhlAn (Version 4.1.1, 11 March 2024) with the mpa_vJan21_CHOCOPhlAnSGB_202103. The functional profiling of the genes, metabolic pathways, and modules was conducted using HUMAnN 3 (HMP Unified Metabolic Analysis Network v3.0) with UniRef90 annotations [19]. All the analysis tools were applied using developer-recommended parameters.

To reduce the technical heterogeneity between samples collected at different time points, MMUPHin batch effect adjustment was applied with maternal BMI as a covariate.

Statistical processing and data visualization were conducted using Python 3.9, with scientific computation and data analysis libraries including NumPy, Pandas, SciPy, statsmodels, scikit-bio, matplotlib, and others. Additional calculations related to metagenomic and functional biomarker identification and differential analysis were performed using LEfSe. Alpha and beta biodiversity assessment was conducted using the Shannon index and Bray–Curtis dissimilarity metric (between groups). Ordination of the functional data was carried out using the Jensen–Shannon metric.

### 2.5. Antibiotic Resistance Gene Screening

Antibiotic resistance gene (ARG) identification was performed by comparing the assembled metagenomic sequences (metaSPAdes) with the NCBI, CARD, Resfinder, and ARG-ANNOT databases using ABRicate software. Only sequences with >90.0% coverage and >70.0% identity were included in the analysis. Further statistical analysis and result visualization were based on the CARD database data, which provided the highest number of identified ARGs with higher sequence coverage and identity compared to the other databases.

## 3. Results

The recruitment of volunteers for the study and control groups was conducted at Perinatal Center No. 2 in Astana from February 2023 to February 2024. The samples for shotgun sequencing and metagenomic analysis were collected at the following time points: 3 DB—first 72 h of life, 1 MB—after 30 days of life, 3 MB—after 3 months of life, and 6 MB—after 6 months.

The study cohort comprised a total of 48 participants divided into two groups: 12 mothers with class 2 and 3 obesity (BMI 37.9 ± 1.58 kg/m^2^ [95.0% CI: 36.05–40.0]) and their 12 infants. The control group consisted of 12 women with normal weight (BMI 27.7 ± 2.3 kg/m^2^ [95.0% CI: 24.3–31.9]) and their infants. The mean age of the participants was 33.3 ± 5.33 years (range 19–42 years), with no statistically significant intergroup differences (*p* = 0.43). The median parity was comparable between the groups: 3.0 ± 1.3 and 3.0 ± 1.15, respectively (*p* = 0.52). Analysis of the obstetric parameters revealed that gestational age at delivery in the obese mothers group was 278.8 ± 2.6 days, compared to 282.0 ± 3.4 days in the control group (*p* = 0.12). The proportions of vaginal deliveries were 33.0% (4/12) and 83.0% (10/12), respectively (*p* = 0.34). The assessment of the neonatal anthropometric parameters showed that the mean birth weight was 3843.0 ± 554.2 g in the maternal obesity group and 4167.5 ± 282.4 g in the control group (*p* = 0.24). Neonatal body length was 55.8 ± 1.5 cm and 57.0 ± 1.79 cm, respectively (*p* = 0.26).

During the follow-up period, by six months of age, comparable weight gain was observed in both groups (Table 1), with a similar trend noted for body length.

Gender distribution was characterized by a predominance of males in both groups: the female/male ratio was 3/9 in the study group and 5/7 in the control group (*p* = 0.67). At each analysis stage, some samples were excluded due to quality control failure or insufficient read counts, resulting in varying final sample numbers across analytical phases.

At each stage of the analysis, some samples were excluded due to non-compliance with quality control requirements or insufficient number of reads, resulting in different results in the final quantities.

For the analysis of gut microbiota at each temporal stage, we applied shotgun sequencing to a subset of fecal samples (N = 77, 64.1% of the initial cohort). The shotgun sequencing analysis yielded a total of 6,670,000,000 paired reads, with a mean of 35,000,000 sequences per sample.

**Figure 1:**
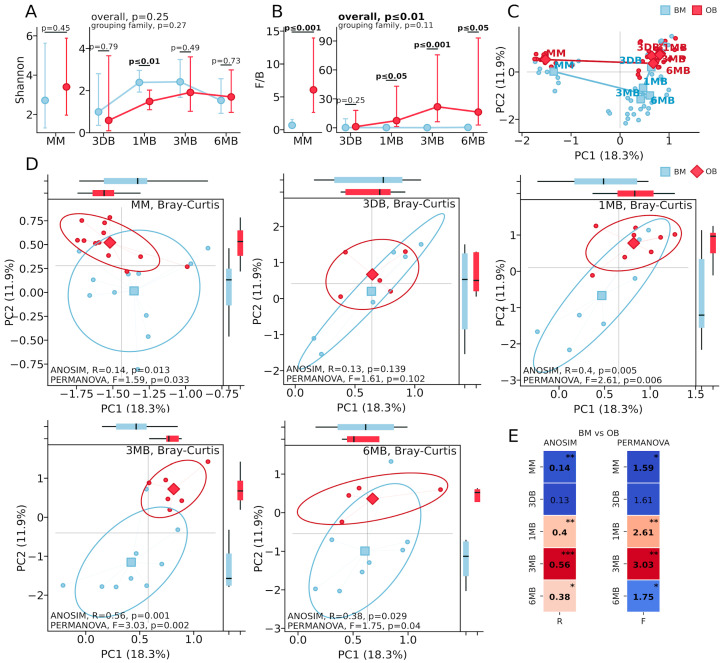
Analysis of gut microbiota alpha and beta diversity in mother–infant pairs in the context of maternal obesity. (**A**) Alpha diversity dynamics (Shannon index) in BM and OB groups. *p*-values are indicated for each time point on the graph. (**B**) Firmicutes/Bacteroidetes (F/B) ratio across time points. (**C**) Principal Coordinates Analysis (PCoA) of all samples based on Bray–Curtis distance. (**D**) Microbiota beta diversity (PCoA based on Bray–Curtis distance) at different time points: maternal samples (MM, BM, n = 12, OB, n = 12), at 0–3 days (3 DB, BM, n = 6, OB, n = 5), 1 month (1 MB, BM, n = 7, OB, n = 8), 3 months (3 MB, BM, n = 9, OB, n = 6), and 6 months (6 MB, BM, n = 8, OB, n = 4) after birth. Blue indicates BM group samples; red indicates OB group samples. Confidence ellipses represent 95.0% confidence intervals. (**E**) Statistical significance of differences between groups was assessed using ANOSIM and PERMANOVA tests with visualization of R and F values. * *p* < 0.05, ** *p* < 0.01, *** *p* < 0.001.

The microbiota diversity analysis revealed distinct patterns between the OB and BM groups. Alpha diversity, assessed using the Shannon index (Figure 1A), showed a significant reduction in the OB group only at one month of age (*p* ≤ 0.01), while differences at other time points were not statistically significant (MM: *p* = 0.45; 3 DB: *p* = 0.79; 3 MB: *p* = 0.49; 6 MB: *p* = 0.73). The Firmicutes/Bacteroidetes ratio (Figure 1B) was significantly elevated in the OB group, beginning with maternal samples (*p* ≤ 0.001), reaching maximum differences at 3 months (*p* ≤ 0.0001), and maintaining differences at 6 months (*p* ≤ 0.01). Principal Coordinates Analysis (PCoA) based on the Bray–Curtis distance (Figure 1C), demonstrated the sequential formation of microbial communities from the maternal samples through the meconium to the mature infant microbiota, with a clear separation of samples both across time points and between the OB and BM groups.

Analysis of the microbiota beta diversity based on the Bray–Curtis distance revealed dynamic differences in the gut microbiota structure among infants born to mothers with obesity (Figure 1). Significant differences between the groups were detected in the maternal gut microbiota samples (Figure 1D, MM: ANOSIM: R = 0.14, *p* = 0.013; PERMANOVA: F = 1.59, *p* = 0.033). No significant differences in the microbial community structure were observed in the first-pass meconium samples (Figure 1D, 3 DB: ANOSIM: R = 0.13, *p* = 0.139; PERMANOVA: F = 1.61, *p* = 0.102). By one month of age (Figure 1D, 1 MB), substantial differences in the microbiota composition between the groups had developed (ANOSIM: R = 0.4, *p* = 0.005; PERMANOVA: F = 2.61, *p* = 0.006), reaching maximum divergence at three months of age (Figure 1D, 3 MB: ANOSIM: R = 0.56, *p* = 0.001; PERMANOVA: F = 3.03, *p* = 0.002). By six months (Figure 1D, 6 MB), differences between the groups persisted but were less pronounced, though remaining statistically significant (ANOSIM: R = 0.38, *p* = 0.029; PERMANOVA: F = 1.75, *p* = 0.04).

Principal Coordinates Analysis (PCoA) based on the Bray–Curtis distance (Figure 1C) and beta diversity visualization at individual time points (Figure 1D) demonstrated clear a separation of the microbial communities between the OB and BM groups, which was particularly pronounced at 1 and 3 months of age. The first two principal components explain 18.3% and 11.9% of the total variance, respectively. The statistical metrics heat map (Figure 1E) further corroborates the pronounced differences between groups at the 1 MB and 3 MB time points, with a slight attenuation at 6 MB while maintaining statistical significance.

**Figure 2:**
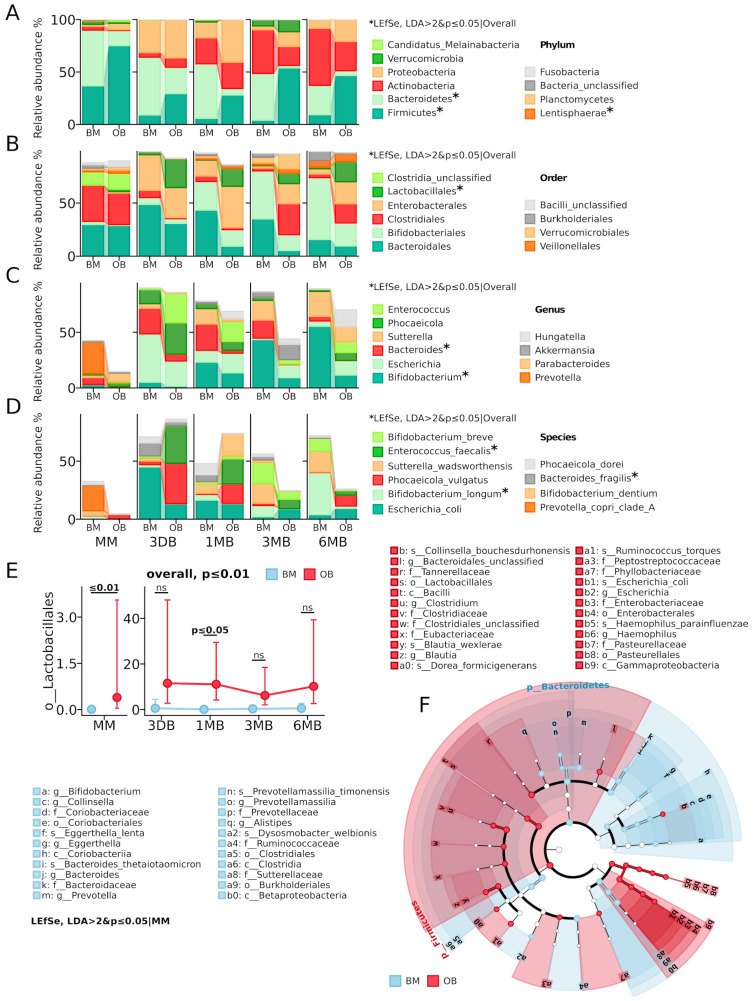
Analysis of gut microbiota taxonomic composition in mother–infant pairs with normal weight (BM) and obesity (OB). (**A**) Taxonomic composition at the phylum level, showing the distribution of major bacterial phyla. (**B**) Taxonomic composition at the order level. (**C**) Microbiota composition at the genus level. (**D**) Species composition of the microbiota. (**E**) Dynamics of relative abundance of *Lactobacillales* in the study groups (*p* = 0.005). (**F**) The cladogram reflects taxonomic differences between groups based on LEfSe analysis (LDA > 2, *p* ≤ 0.05); green indicates taxa predominant in the normal weight group (BM), red indicates taxa predominant in the obesity group (OB). Statistically significant differences are marked with asterisks (*: *p* ≤ 0.05, “ns”—not significant). Time points: MM—maternal feces, 3 DB—0–3 days, 1 MB—1 month, 3 MB—3 months, 6 MB—6 months.

Taxonomic analysis revealed substantial differences in the microbiota composition between the OB and BM groups (Figure 2). At the phylum level (Figure 2A), the maternal microbiota showed significant differences in the abundance of *Firmicutes* (current name *Bacillota*) on average (OB: 75.2% vs. BM: 36.9%) and *Bacteroidetes* (current name *Bacteroidota*) (OB: 14.3% vs. BM: 53.1%). The OB group also showed elevated levels of *Proteobacteria* (current name *Pseudomonadota*) (6.0% vs. 1.8% in BM) and *Candidatus_Melainabacteria* (1.7% vs. 0.3% in BM). In the meconium samples (3 DB, Figure 2D), Proteobacteria dominated in both groups (OB: 36.5% vs. BM: 30.9%), primarily due to *E. coli*, with the OB group characterized by increased *Firmicutes* (29.5% vs. 9.1% in BM) and decreased *Bacteroidetes* (25.0% vs. 55.0% in BM). By one month of age (1 MB), a significant increase in *Actinobacteria* (current name *Actinomycetota*) was observed in both groups (OB: 25.0% vs. BM: 24.9%), with the OB group maintaining elevated levels of *Proteobacteria* (40.6% vs. 14.9%) and *Firmicutes* (28.1% vs. 5.9%), while *Bacteroidetes* predominated in the BM group (51.9% vs. 6.2%). The most pronounced intergroup differences were recorded at three months of age (3 MB), when *Firmicutes* dominated in the OB group (53.9% vs. 4.1%) and *Verrucomicrobia* (current name *Verrucomicrobiota*) proportion increased (11.6% vs. 5.8%), while the BM group was characterized by high levels of *Bacteroidetes* (44.5% vs. 2.3%) and *Actinobacteria* (41.7% vs. 18.2%). By six months (6 MB), the difference profile persisted: a predominance of *Firmicutes* (46.7% vs. 9.3%) and elevated *Proteobacteria* (20.8% vs. 8.3%) in the OB group, with *Actinobacteria* dominating in the BM group (54.4% vs. 27.9%).

At the order level (Figure 2B,E), a significant increase in *Lactobacillales* abundance was observed in the OB group (*p* ≤ 0.01), with maximum differences in the maternal samples (*p* ≤ 0.01) and after 1 month of early infant life (*p* ≤ 0.05). A consistent decrease in the relative abundance of *Bacteroidales* and increase in *Clostridiales* (current name *Eubacteriales*) was also noted in the OB group throughout the observation period. At the genus and species levels (Figure 2C,D), the OB group showed elevated levels of *Enterococcus* faecalis (*p* ≤ 0.05), while the BM group was characterized by a predominance of *Bifidobacterium* (*p* ≤ 0.05) representatives, especially *B. longum* (*p* ≤ 0.05) and *Bacteroides* (*p* ≤ 0.01). These results indicate persistent differences in taxonomic composition between the groups at all taxonomic levels, starting from the maternal microbiota and persisting throughout the first six months of life.

**Figure 3:**
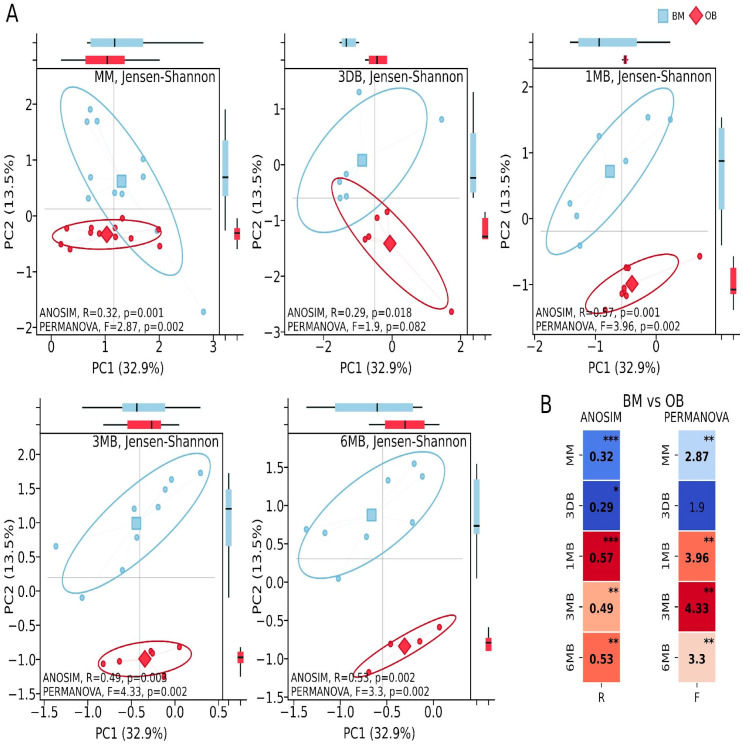
PCoA analysis of microbiota metabolic pathways during early postnatal period dynamics. (**A**) PCoA plots showing separation of metabolic pathways in maternal samples (MM, BM, n = 12, OB, n = 12), at 0–3 days (3 DB, BM, n = 6, OB, n = 5), 1 month (1 MB, BM, n = 7, OB, n = 8), 3 months (3 MB, BM, n = 9, OB, n = 6), and 6 months (6 MB, BM, n = 8, OB, n = 4) after birth. Blue indicates BM group samples; red indicates OB group samples. Confidence ellipses represent 95.0% confidence intervals. Statistical significance of differences between groups was assessed using ANOSIM and PERMANOVA tests. PC1 and PC2 are principal components explaining 32.9% and 13.5% of total variance, respectively. (**B**) Heatmap summary of ANOSIM (R values) and PERMANOVA (F values) test results comparing BM vs OB groups across all timepoints. Asterisks indicate statistical significance levels (* *p* < 0.05, ** *p* < 0.01, *** *p* < 0.001).

Metabolic activity analysis identified 415 metabolic pathways, of which 43 showed statistically significant differences between the study groups (*p* ≤ 0.05 and non-overlapping 95.0% CI and effect difference between means > 0.3). Pathway identification was conducted using the curated MetaCyc database (Version 28.5). Principal Coordinates Analysis (PCoA) of the metabolic pathways showed substantial intergroup differences in the maternal samples between the OB and BM groups (ANOSIM: R = 0.32, *p* = 0.001; PERMANOVA: F = 2.87, *p* = 0.002). Considerable differences were found in the first-pass meconium samples (ANOSIM: R = 0.29, *p* = 0.018; PERMANOVA: F = 1.9, *p* = 0.082). In the infants, significant differences were observed during periods of intensive growth and increasing microbial diversity: after one month of life (ANOSIM: R = 0.57, *p* = 0.001; PERMANOVA: F = 3.96, *p* = 0.002), three months (ANOSIM: R = 0.49, *p* = 0.003; PERMANOVA: F = 4.33, *p* = 0.002) and six months (ANOSIM: R = 0.53, *p* = 0.002; PERMANOVA: F = 3.33, *p* = 0.002) (Figure 3).

**Figure 4:**
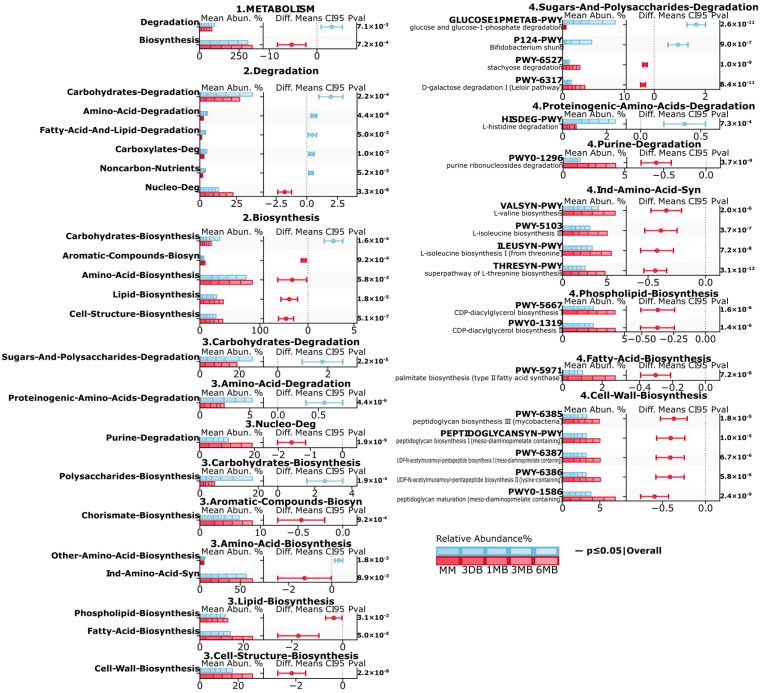
Multi-level analysis of gut microbiota metabolic pathways in mother–child pairs with obesity and normal weight. Hierarchical analysis of metabolic pathway abundance and differences between obesity (OB) and normal weight (BM) groups. Data are presented at four organizational levels (1–4) according to the MetaCyc database. Each pathway displays mean abundance (Mean Abun. %, left), between-group differences with 95.0% confidence intervals (Diff. Means CI95, middle), and significance value (Pval, right). Time points are color-coded: MM—maternal feces, 3 DB—0–3 days, 1 MB—1 month, 3 MB—3 months, 6 MB—6 months. Red error bars indicate significant differences (*p* ≤ 0.05). Negative Diff. Means CI95 values indicate higher abundance in the OB group; positive values indicate higher abundance in the BM group.

Metagenomic analysis revealed substantial differences in the microbiota metabolic pathways between the infants of mothers with obesity (OB) and normal weight (BM) (Figure 4). At the first level (1. METABOLISM), a significant decrease in the degradation (*p* ≤ 0.01) and increase in the biosynthesis (*p* ≤ 0.001) pathways were observed in the OB group. Second-level analysis revealed a significant decrease in the carbohydrate (*p* ≤ 0.001, amino acid (*p* ≤ 0.001), carboxylate (*p* ≤ 0.01), degradation pathways in the OB group, increases in lipid biosynthesis (*p* ≤ 0.001), cellular structure biosynthesis (*p* ≤ 0.001), and amino acid biosynthesis (*p* ≤ 0.01), and elevated activity in the aromatic compound biosynthesis pathways (*p* ≤ 0.001). At the third level, the BM group showed increases in carbohydrate and polysaccharide degradation (*p* ≤ 0.001) and proteinogenic amino acid degradation (*p* ≤ 0.001), while purine degradation (*p* ≤ 0.001), as well as phospholipid biosynthesis (*p* ≤ 0.05), fatty acid biosynthesis (*p* ≤ 0.001), and cell wall component biosynthesis (*p* ≤ 0.001), showed increases in the OB group.

Detailed fourth-level analysis revealed significant increases in the specific glucose degradation (GLUCOSE1PMETAB-PWY, *p* ≤ 0.001), Bifidobacterium shunt (P124-PWY, *p* ≤ 0.001), and histidine degradation (HISDEG-PWY, *p* ≤ 0.001) pathways in the BM group. The OB group also showed increased branched-chain amino acid biosynthesis pathways (VALSYN-PWY, ILEUSYN-PWY, *p* ≤ 0.001), CDP-diacylglycerol biosynthesis (PWY-5667, PWY0-1319, *p* ≤ 0.001), and peptidoglycan biosynthesis (PWY-6385, PEPTIDOGLYCANSYN-PWY, *p* ≤ 0.001). These differences indicate substantial restructuring of the microbiota metabolic potential in the OB group, characterized by increased activity in anabolic processes, particularly in amino acid, and the lipid metabolism pathways.

Metabolomic analysis of gut microbiota revealed significant differences between infants of mothers with obesity (OB) and normal weight (BM). Fecal pH analysis showed dynamic differences between groups (Supplementary Table S1). Mothers in the OB group showed significantly lower pH (6.2 ± 0.23 vs. 6.88 ± 0.24, *p* < 0.001). No significant differences were found in meconium samples (3 DB, *p* = 0.2) and at one month (1 MB, *p* = 0.39). By three months, the OB group showed decreased pH (5.61 ± 0.35 vs. 6.35 ± 0.36, *p* ≤ 0.01), however, by six months, a significant increase in pH compared to the BM group was observed (6.31 ± 0.97 vs. 5.54 ± 0.11, *p* ≤ 0.05). Correlation analysis between metabolic pathways and pH reduction (Supplementary Table S1) showed strong negative correlations with fatty acid biosynthesis (r = −0.497, *p* < 0.001) and polyprenol biosynthesis (r = −0.480, *p* < 0.001), moderate positive correlateions with coenzyme A biosynthesis (r = 0.353, *p* < 0.01) and chorismate biosynthesis (r = 0.327, *p* < 0.01), and a weak negative correlation with other amino acid biosynthesis (other than those used for protein synthesis) (r = −0.279, *p* < 0.05).

**Figure 5:**
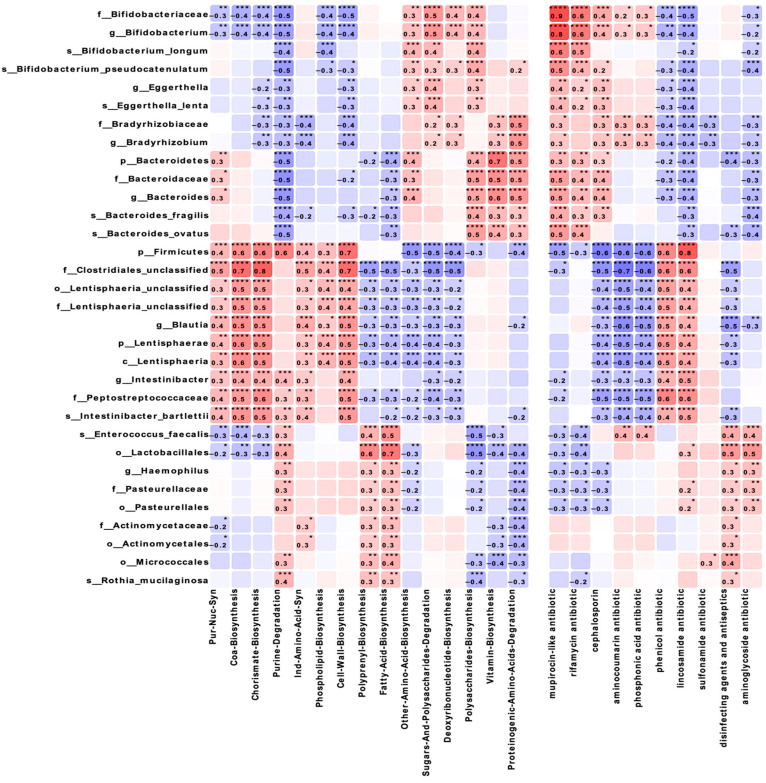
Correlation analysis between microbial taxa and metabolic pathways/antibiotic resistance genes. Heatmap showing Spearman correlation coefficients between bacterial taxa (rows) and metabolic pathways and antibiotic resistance genes (columns) across all samples. Red indicates positive correlations; blue indicates negative correlations. The intensity of color corresponds to correlation strength. Statistical significance levels are indicated by asterisks: * *p* ≤ 0.05, ** *p* ≤ 0.01, *** *p* ≤ 0.001, **** *p* ≤ 0.0001.

Correlation analysis of the taxonomic composition and functional characteristics of the microbiota (Figure 5) revealed that *Lactobacillales* showed a strong positive correlation with fatty acid biosynthesis (r = 0.7, *p* ≤ 0.0001) and a negative correlation with polysaccharide biosynthesis (r = −0.5, *p* ≤ 0.001). Firmicutes demonstrated significant correlations with coenzyme A biosynthesis (r = 0.6, *p* ≤ 0.0001) and chorismate (r = 0.6, *p* ≤ 0.0001), as well as with lincosamide resistance genes (r = 0.8, *p* ≤ 0.0001). *Bifidobacteriaceae* showed negative correlations with purine degradation and cell wall biosynthesis (r = −0.5, *p* ≤ 0.0001), while *Bacteroidetes* demonstrated moderate positive correlations with amino acid, polysaccharide biosynthesis, and proteinogenic amino acids degradation (r = 0.4–0.5, *p* ≤ 0.01) and a strong positive correlation with vitamin biosynthesis (r = 0.7, *p* ≤ 0.01).

## 4. Discussion

This study presents a comprehensive analysis of how maternal obesity affects the development of the infant gut microbiome during the critical first 6 months of life. Our shotgun metagenomic approach revealed taxonomic alterations and important functional changes in metabolic pathways that may have long-term consequences for infant health. The dynamic patterns observed across multiple time points highlight critical windows of microbiome development where maternal obesity exerts its most potent influence.

In this study, we present a comprehensive dynamic picture of intestinal microbiome formation in infants born to obese mothers during the first 6 months of life. While previous studies have demonstrated general changes in infant microbiota associated with maternal obesity [10,13,20], our work extends these observations by demonstrating not only taxonomic changes but also their functional metabolic consequences using whole metagenomic sequencing. The significant decrease in alpha diversity in infants from the OB group at one month of age indicates a critical window in microbiome establishment, which is consistent with the concept of early metabolic programming [21]. A comparison with longitudinal studies of the infant microbiome, such as TEDDY [22], shows that the temporal patterns of the microbiota we identified represent distinct developmental trajectories. The observed elevated *Firmicutes/Bacteroidetes* ratio in the OB group, which is particularly pronounced at 3 months (*p* ≤ 0.001), is of particular interest. Unlike previous studies [23,24], we observe that this imbalance forms dynamically, beginning with meconium microbiota and reaching a maximum at 3 months of infant life. This fact may indicate vertical transmission of the microbiome from mother to child, which is supported by studies by Ferretti et al. [15]. The depletion of the relative abundance of *Bacteroides* and *Bifidobacterium longum* in the OB group has potentially serious implications for future metabolic health. Vatanen et al. [25] showed that *Bacteroides* play a key role in degrading complex polysaccharides and maintaining the intestinal barrier, and their deficiency may contribute to future metabolic disorders.

An important aspect is the identification of the substantial reprogramming of the metabolic potential of the microbiome in the OB group. Unlike other studies focused predominantly on taxonomic changes [13,26], we found complex changes in metabolic pathways that may explain the mechanisms of maternal obesity’s influence on children’s health. The decreased presence of carbohydrate degradation pathways, amino acids, and carboxylates, with a simultaneous increase in lipid biosynthesis pathways and cellular structures in our study cohort, suggests early metabolic programming. These changes are consistent with a study by Costa et al. [17], which showed the effect of maternal obesity on the expression of genes associated with the mitochondrial and lipid metabolism in the umbilical vein endothelial cells of infants, but we demonstrate for the first time that similar changes are reflected in the metabolic potential of the intestinal microbiota. These changes can potentially contribute to programming the child’s metabolic status through the production of bioactive lipids and modulation of host signaling pathways.

The identified strong correlations between the taxonomic groups and metabolic pathways reveal new potential aspects in understanding the functional ecology of the microbiome in maternal obesity. The positive correlation of *Lactobacillales* with fatty acid biosynthesis and negative correlation with polysaccharide biosynthesis may reflect compensatory mechanisms aimed at maintaining metabolic homeostasis [27,28]. The correlations of *Firmicutes* with genes for resistance to lincosamides and the phenicol resistance gene are of particular interest in the context of the growing problem of antibiotic resistance. Compared to data on the resistome of the intestinal microbiota (Gasparrini et al., 2019) [29], our study shows that maternal obesity may influence the formation of the resistome in infants, which has potentially important clinical implications. The dynamics of fecal pH, reflecting the metabolic activity of the microbiota, in our opinion, deserve special attention. Increased fecal acidity in mothers in the OB group and infants at 3 months may be associated with the increased production of short-chain fatty acids. This is consistent with data on the influence of maternal obesity on the metabolic activity of newborn microbiota [20], but our study demonstrates dynamic changes in this parameter for the first time during the first 6 months of life.

The identified specific time windows (1 and 3 months) may represent the optimal periods for therapeutic intervention. Unlike previous works proposing general strategies for microbiome modulation [30], our data on specific metabolic pathways allow the development of more targeted approaches. The strong correlations between specific taxa and metabolic functions provide new targets for probiotic and prebiotic therapy. For example, stimulation of *Bacteroidetes* and *Bifidobacterium* representatives may help restore the polysaccharide degradation pathways, while the modulation of *Lactobacillales* activity could potentially normalize lipid metabolism. Our study has several limitations to consider when interpreting results. The relatively small sample size (24 mother–infant pairs) and single-center study design may limit result generalizability. The 6-month observation period, while covering a critical microbiome establishment period, does not allow assessment of the long-term consequences of the observed changes. Additionally, the lack of detailed maternal diet composition data during pregnancy and lactation, along with sample loss due to quality control failure, may have affected result completeness. Despite these limitations, the obtained data on taxonomic composition and the metabolic potential differences between the OB and BM groups remain statistically significant and align with other research findings [20], confirming their value and contribution to understanding maternal obesity’s influence on infant microbiome formation. Future studies with larger cohorts, longer observation periods, and the integration of host metabolomic data will allow a more comprehensive characterization of the interaction between maternal obesity, the infant microbiome, and long-term health consequences.

## 5. Conclusions

These results demonstrate that maternal obesity induces profound changes in the metabolic function of the infant gut microbiome, mainly through a shift from carbohydrate degradation to enhanced lipid biosynthesis. This metabolic reprogramming may shed light on the mechanism of the long-term programming of metabolic health in children born to obese mothers. The identified specific time windows at one and three months of infant life represent critical periods during which the impact of maternal obesity on the developing microbiome is most pronounced. The strong correlations we observed between the bacterial taxa and specific metabolic functions provide new potential targets for preventive strategies. These include probiotic interventions to correct the Firmicutes/Bacteroidetes balance, prebiotic approaches to stimulate the polysaccharide degradation pathways, and metabolic support to normalize the altered lipid metabolism in infants born to mothers with obesity.

These findings contribute to our understanding of the intergenerational transmission of obesity risk and suggest novel approaches for early intervention that may help mitigate the long-term health consequences of maternal obesity for offspring.

## Figures and Tables

**Table 1:** Cohort characteristics.

Indicators	Obese	Normal Weight Control	Total	*p*-Value, Obese vs. Normal Weight Control
Mother, n	12	12	24	
Maternal age (year) ^1^	33.9 ± 5.43 (19–42)	32.7 ± 5.41 (21–42)	33.3 ± 5.33 (19–42)	0.43 ^a^
Average number of births ^1^	3.3 ± 1.3 (1–6)	3.0 ± 1.15 (1–5)	3.1 (1–6)	0.52 ^a^
Maternal BMI (kg/m^2^) ^1^	37.9 ± 1.58 (36.05–40)	27.7 ± 2.3 (24.3–31.9)	32.8 ± 5.5 (24.3–40)	≤0.0001 ^b^
Maternal Weight (kg) ^1^	100.6 ± 6.5 (90–110)	73.3 ± 10.9 (60–92)	86.9 ± 16.5 (60–110)	≤0.0001 ^b^
Gestational Age at Birth (days) ^1^	278.8 ± 2.6 (276–283)	282.0 ± 3.4 (278–288)	280.5 ± 3.3 (276–288)	0.12 ^b^
Vaginal Birth, %	33.3	83.3	81.8	0.31 ^c^
Metagenomic profiling samples:				
Newborn, n	5	6	11	
Infant, 1 month, n	8	7	15	
Infant, 3 month, n	6	9	15	
Infant, 6 month, n	4	8	12	
Weight:				
- birth weight (g) ^1^	3843.0 ± 554.2 (3200–4670)	4167.5 ± 282.4 (3670–4370)	4020 ± 417.2 (3200–4670)	0.24 ^b^
- after 1 month (g) ^1^	5000.0 ± 545.1 (4100–5800)	4576.57 ± 493.5 (4136–5200)	4802.4 ± 548.4 (4100–5800)	0.18 ^a^
- after 3 months (g) ^1^	7066.67 ± 833.5 (6000–8000)	6836.89 ± 746.6 (5800–7900)	6930 ± 761.5 (5800–8000)	0.59 ^b^
- after 6 months (g) ^1^	8675.0 ± 950 (7400–9400)	8718.75 ± 915.7 (7000–9500)	8704.16 ± 883.3 (7000–9500)	0.94 ^b^
Length:				
- birth length (cm) ^1^	55.8 ± 1.5 (54–58)	57 ± 1.79 (54–59)	56.45 ± 1.7 (54–59)	0.26 ^b^
- after 1 month (cm) ^1^	56.5 ± 1.8 (53–59)	57.14 ± 3.2 (52–62)	56.8 ± 2.5 (52–62)	0.63 ^b^
- after 3 months (cm) ^1^	62.33 ± 4.5 (55–68)	63.11 ± 2.9 (59–67)	62.8 ± 3.5 (55–68)	0.69 ^b^
- after 6 months (cm) ^1^	69.875 ± 1.7 (68–72)	71.12 ± 4.2 (64–78)	70.7 ± 3.6 (64–78)	0.59 ^b^
Female/Male Infants (n/n)	3/9	5/7	8/16	0.67 ^c^
Apgar score (A/S)	8/9	8/9	8/9	-

^1^ Mn ± Sd, (min–max). ^a^ Mann–Whitney U rank test. ^b^ Ind. *t*-test. ^c^ Fisher’s exact test.

## Data Availability

The data from this study have been deposited in the NCBI BioProject database under the accession number PRJNA949528; PRJNA1217583. All datasets are publicly accessible through this repository.

## Supplementary Materials

The following supporting information can be downloaded at: https://www.mdpi.com/article/10.3390/biomedicines13040838/s1, Table S1: Dynamic changes in fecal pH values and their correlation with metabolic pathways in infants from BM and OB groups across different timepoints.
